# Assessment of functioning using the WHODAS 2.0 among people with myasthenia gravis-associated disability: a nationwide follow-up study

**DOI:** 10.1186/s12889-025-23328-5

**Published:** 2025-07-02

**Authors:** Jia-Pei Hong, Chih-Hong Lee, Chien Tai Hong, Lung Chan, Chen-Chih Chung, Prangthip Charoenpong, Hou-Chang Chiu, Tsan-Hon Liou

**Affiliations:** 1 Department of Physical Medicine and Rehabilitation, Taipei Medical University Shuang Ho Hospital, Taipei, Taiwan; 2 Department of Neurology, Chang Gung Memorial Hospital Linkou Medical Center, Chang Gung University College of Medicine, Taoyuan, Taiwan; 3 Taipei Neuroscience Institute, Taipei Medical University, Taipei, Taiwan; 4 Department of Neurology, Taipei Medical University-Shuang Ho Hospital, New Taipei City, Taiwan; 5 Department of Neurology, School of Medicine, College of Medicine, Taipei Medical University, Taipei, Taiwan; 6 Division of Pulmonary, Critical Care, and Sleep Medicine, Department of Internal Medicine, Baylor College of Medicine, Houston, TX USA; 7 Department of Neurology, Shin Kong Wu Ho-Su Memorial Hospital, Taipei, Taiwan; 8 Department of Physical Medicine and Rehabilitation, School of Medicine, College of Medicine, Taipei Medical University, Taipei, Taiwan; 9 Department of Physical Medicine and Rehabilitation, WanFang Hospital, Taipei Medical University, No.111, Sec. 3, Xinglong Rd., Wenshan Dist, Taipei City, 116 Taiwan

**Keywords:** Myasthenia Gravis, Disability evaluation, International classification of functioning, disability, and health

## Abstract

**Background:**

Advancements in treatment have increased the life expectancy of patients with Myasthenia Gravis (MG), yet the understanding of functional changes in these individuals remains limited. In our study, we explored the functional abilities of individuals with MG using the World Health Organization Disability Assessment Schedule 2.0 (WHODAS 2.0).

**Methods:**

This observational study analyzed data from 286 patients with MG, acquired from the Data Bank of Persons with Disabilities (TDPD) in Taiwan between July 11, 2012, and December 31, 2021. Participants were diagnosed with MG after the acute phase. The functional disability outcome was assessed using the WHODAS 2.0. Due to non-normal distribution of variables, we used the Wilcoxon rank sum and chi-square tests for group comparisons by age. Longitudinal associations between functional changes and demographic characteristics were assessed using generalized estimating equations (GEE), adjusting for age, sex, educational level, and respiratory dysfunction.

**Results:**

The average follow-up period was three years. At the initial evaluation, younger individuals (< 65 years) generally demonstrated less severe disability across most functional domains, with the exception of social participation. Patients with a single disability in a specific function performed better than those with multiple disabilities. The provision of technical aids or other environmental interventions resulted in significant functional improvements across all domains. During follow-up, significant functional improvements were observed, especially among patients residing in institutions, those without respiratory disabilities, and those with reduced mobility at the time of initial assessment. The GEE analysis indicated that younger patients with MG experienced functional changes comparable to older patients. Notably, respiratory disability was identified as a key factor contributing to cognitive decline. Additionally, only 10.5% of patients aged 18–64 were employed at the time of the study.

**Conclusions:**

Our findings highlight the complexity of disabilities associated with MG, with social participation emerging as a particularly vulnerable domain. Although younger patients initially exhibited better functional performance, their long-term functional changes were comparable to those observed in older patients. Given the impact of functional limitations on employment, future research may focus on return-to-work interventions, as labor market participation plays a crucial role in fostering social engagement for individuals with MG.

## Background

Myasthenia gravis (MG) is a rare autoimmune disease but is the most common condition affecting the neuromuscular junction (NMJ) in skeletal muscle, with a worldwide prevalence of approximately 20 per 100,000 [1]. Manifestations include fluctuating fatigue and varied degrees of weakness affecting the upper and lower limbs, trunk, eyes and face, bulbar, and respiratory muscles. MGs may present with eyelid ptosis, diplopia, limb weakness, difficulty swallowing, or impaired speaking and breathing. Spontaneous remission is infrequent and occurs primarily within the first 3 years of disease onset [2]. Despite advancements in treatment, MG currently lacks a definitive cure, with interventions such as acetylcholinesterase inhibitors and disease-modifying immunosuppressors offering only transient symptom relief or modulation of autoimmune responses [3].

As the life expectancy of MG patients has improved, research has increasingly focused on understanding the impact of MG on quality of life. A previous study suggested a potential association between the severity of MG and functional status, warranting further investigation, particularly given the wide clinical spectrum of MG—ranging from mild symptoms to life-threatening manifestations, such as myasthenic crises [4]. While certain social determinants, particularly employment status, have demonstrated associations with improved quality of life among MG patients, there remains a paucity of research exploring the broader spectrum of social consequences and functional impairments intrinsic to MG [5].

Current functional impairment measures lack consideration of the environmental factors influencing daily activity execution [6]. Recognizing this limitation, the World Health Organization (WHO) introduced the WHO Disability Assessment Schedule (WHODAS 2.0). Rooted in the conceptual framework of the International Classification of Functioning, Disability, and Health (ICF), this instrument comprehensively evaluates functional disability, covering domains such as work-related disability and community participation. Given its versatility and applicability in assessing health status and disability across diverse populations, the WHODAS 2.0 has emerged as a robust tool for comprehensively evaluating the impact of MG, making it well-suited for the objectives of this study [7].

Despite scant information on long-term changes in functional impairments among MG patients, our study is attempts to fill this gap. The prevalence of MG in Taiwan is comparable to that in other populations [8]; therefore, we conducted a nationwide, retrospective database study to analyze the functional outcomes of MG in the context of a 9-year timeframe. We aimed to explore the functional features of MG, hypothesizing that the WHODAS 2.0 could capture long-term functional changes and environmental factors in MG patients.

## Methods

### Participants and data collection

This study was performed in line with the principles of the Declaration of Helsinki. Because this is a secondary data analysis study and the data were analysed anonymously, informed consent was not required. This study was approved by the Joint Institutional Review Board of Taipei Medical University (N202107095).

Since 2012, Taiwan mandated that the assessment of eligibility for disability benefits must be based on the ICF framework. Anyone who is susceptible to dysfunction or a disability because of their disease can apply for a disability assessment. The assessments were performed when the patient had a stationary status of disability, usually during 4 to 6 months after the acute period. The re-evaluation time of disability assessment depends on the validity of disability benefits, usually after 1–5 years of the initial evaluation. After assessment, people who are eligible for disability benefits can receive welfare and service support from the government, and their data will be included in the Taiwan Data Bank of Persons with Disability (TDPD). The TDPD is maintained by the Taiwan Ministry of Health and Welfare, and the original identification number of each patient is deidentified. The database comprises information on 978,038 individuals who had disabilities in the 2012–2020 fiscal years.

In this study, we collected data from TDPD between July 11, 2012, and December 31, 2021. We included individuals with MG diagnosed according to the *International Classification of Diseases*, *Ninth Revision*, *Clinical Modification* codes ICD-9-CM (code 358) and ICD-10-CM (G70) when they applied for a disability assessment for the first time. The enrolled participants were assessed twice. We selected the last evaluation during our study period as the 2nd assessment. People younger than 18 years of age or loss of follow-up were excluded. Initially, 287 individuals met our inclusion criteria. We excluded individuals aged < 18 years. The final sample comprised 286 participants.

### Patient and public involvement

There was no patient involvement in this study other than that required for disability assessment during visits to homes or institutions.

### Measurement tools

Each assessment was performed in two separate sessions through a specialized disability evaluation system known as the Disability Eligibility Determination Scale 2012 (DES-2012). DES-2012 was developed according to ICF concepts and has been used in Taiwan to assess disability since July 2012 [9]. The first session was conducted by clinical physicians for diagnosing and classifying the impairment aspects, specifically the ICF categories of body functions (b codes) and structures (s codes). Categories from domains of body functions, body structures have been used at the second level (e.g. b730, muscle power functions). Each ICF category was rated with appropriate qualifiers that indicate the extent to which impairments, limitations and restrictions to full functioning are observed. No a priori selection of categories was made. The severity of impairment was determined using the concept of the highest qualifier of the b (b710, Mobility of joint functions; b730, Muscle power functions; b735, Muscle tone functions) codes of ICF. Qualifiers were rated when adequate information was available to describe mild (qualifier 1), moderate (qualifier 2), severe (qualifier 3) or complete (qualifier 4) impairments. If no problem was detected for a specific category, qualifier 0 (no problem) was applied. In DES-2012, considering b730 Muscle power functions as an example, patients with the b730.1 code are classified as having mild impairment.

In DES-2012, the eight disability types are classified according to body functions: [1] Chap. 1 Mental Functions & Structures of the Nervous System; [2] Chap. 2 Sensory Functions and Pain: The Eye, Ear and Related Structures; [3] Chap. 3 Functions & Structures of/involved in Voice and Speech; [4] Chap. 4 Functions & Structures of/Related to the Cardiovascular Hematological, Immunological and Respiratory Systems; [5] Chap. 5 Functions and Structures of/Related to Digestive, Metabolic and Endocrine Systems; [5] Chap. 6 Functions & Structures of/related to the Genitourinary and Reproductive Systems; [7] Chap. 7 Neuromusculoskeletal and Movement-related Functions and Structures; [8] Chap. 8 Functions and Related Structures of Skin. Patients with two or more impairments in body functions were classified as having multiple disabilities.

The second session included the Chinese version of the WHODAS 2.0 and the ICF categories regarding environmental factors (e codes), which were measured by special testers, such as physical therapists, occupational therapists, and social workers. The validity and reliability of this version of the WHODAS 2.0 have been reported in several studies [10]. The WHODAS 2.0 comprises 36 items and evaluates participants’ functions in six domains: [1] cognition (6 items), which assesses communication and thinking activities, including concentration, memory, problem solving, learning, and communication; [2] mobility (5 items), which assesses activities such as standing, moving inside the home, leaving the home, and walking long distances; [3] self-care (4 items), which assesses hygiene, dressing, eating, and alone time; [4] getting along with people (5 items), which assesses interactions with other people and difficulties that may be encountered in this domain due to health conditions; [5] life activities (8 items), which assesses difficulty in performing daily activities (i.e., those people perform most days, including those associated with domestic responsibilities, leisure, work, and school); and [6] participation (8 items), which assesses social dimensions, such as community activities, barriers and hindrances in one’s daily environment, and problems with other issues, such as maintaining personal dignity. On the basis of each participant’s status over the 30 days preceding the administration of the WHODAS 2.0, the responses to each question were scored on a 5-point Likert-type scale (0 = “no difficulty”; 1 = “mild”; 2 = “moderate”; 3 = “severe”; and 4 = “extreme”) to measure the participants’ difficulty in performing the activities. The obtained results were on a scale from 0 to 100, with higher scores indicating more severe disability (0–4, no difficulty; 5–24, mild difficulty; 25–49, moderate difficulty; 50–95, severe difficulty; 96– 100, extreme difficulty). The missing data fields (e.g., unrated items) have a manually imputed value equal to the domain’s mean [11]. 

Each WHODAS 2.0 item with environmental intervention (performance score) and without any intervention (capability score) were rated. The capability scores indicated the extent of activity limitation as a direct manifestation of the person’s health status without assistance (assistance of another person, equipment, or environmental modification). The performance scores reflected the extent to which participants were restricted in engaging with their real-world environments, even when supported by assistive devices or individuals, such as hearing aids or crutches for locomotion. The relative differences (RDs) for each domain (capability score − performance score) reflect the influence of assistive devices and environment modification on a person’s activities and participation [12]. The sign of an RD indicated the role of the whole environment in daily activity performance as a facilitator (positive RD) or a barrier (negative RD). Understanding RD may guide interventions (e.g., through environmental factors) to improve patients’ performance of daily activities [13].

### Covariates

Demographic data, including sex, age, education level, work status (employed or unemployed), type of residence, urbanization level, and duration of follow-up, were collected from the TDPD. Aging is a key risk factor associated with the severity, baseline comorbidities and treatment-related side effects of MG [14]. Poor neurological outcomes following treatment have been reported in patients with myasthenia gravis (MG) aged 65 years and older [15]. For instance, this age group has been associated with a higher proportion of MG-related mortality. Several studies have used 65 years as a threshold to stratify MG patients into subgroups [16, 17]. Therefore, we dichotomized age groups into 18–64 vs. ≥65 years. Because Taiwan implemented 6 years of compulsory education before 1968, education level was dichotomized into ≤ 6 years or > 6 years of education. In addition, the type of residence was classified as community or institutional, and the urbanization level was classified as rural, suburban, or urban. People who were capable of walking without assistance composed the ambulatory group.

### Statistical analysis

Because the variables were not normally distributed, the Wilcoxon rank sum test and chi-square test were used to compare the medians of the continuous variables and the percentages of the categorical variables in the 18–64 and ≥ 65 years age groups, respectively. The Wilcoxon signed-rank test was used to compare the median gap between the capability and performance scores on the WHODAS 2.0 within each group. To assess the longitudinal associations between functional changes and demographic characteristics, we conducted a series of generalized estimating equation (GEE) analyses and adjusted for confounders, including age, sex, education level and having respiratory dysfunctions. Time–group interaction was also included in the model. We used SAS 9.4 (SAS Institute, Cary, NC, USA) for all the statistical analyses. All reported probabilities (p values) were two-sided, with *p* < 0.05 considered to indicate statistical significance.

## Results

The baseline demographic characteristics of the participants stratified by age group (18–64 vs. ≥65 years) are presented in Table 1. The average age of the included participants was 64.2 years, and 55.6% of the participants were female. Significant differences were observed between the two age groups in employment status (*p* = 0.0002), type of residence (*p* = 0.0298), education level (*p* < 0.0001), type of disability (*p* = 0.034), severity of disability (*p* = 0.0003) and ambulatory status (*p* < 0.001). Among those aged 18–64 years, 10.5% lived in the institution, while 19.7% of those aged ≥ 65 years lived in the institution. The most common types of MG-associated disabilities in the 18–64-year-old age group were dysfunctions in the neuromusculoskeletal system (Chap. 7, 69.4%) and cardiovascular, hematological, immunological and respiratory system disabilities (Chap. 4, 14.2%). In the ≥ 65 years age group, the major types of disabilities were dysfunctions in the neuromusculoskeletal system (Chap. 7, 63.2%) and dysfunctions involving multiple structures (21.7%). The average time between the first and final evaluations was 3 years.

**Table 1:** Demographic data stratified by age

	Total No. (col%) (*n* = 286)	18–64 years No. (col%, row%) (*n* = 134)	65 & 65 years above No. (col%, row%) (*n* = 152)	*p* value^1^
**Age (years)**	64.2 (± 16.4)	49.9 (± 11.7)	76.8 (± 6.9)	< 0.0001
**Sex**				0.0735
Male	127 (44.4%)	52 (38.8%, 40.9%)	75 (49.3%, 59.1%)	
Female	159 (55.6%)	82 (61.2%, 51.6%)	77 (50.7%, 48.4%)	
**Education (years)**				< 0.0001
> 6	139 (48.6%)	119 (88.8%, 85.6%)	20 (13.2%, 14.4%)	
≤ 6	132 (46.2%)	15 (11.2%, 11.4%)	117 (77.0%, 88.6%)	
missing	15 (5.2%)	0 (0.0%, 0.0%)	15 (9.9%, 100.0%)	
**Work Status**				0.0002
Employment	15 (5.2%)	14 (10.5%, 93.3%)	1 (0.7%, 6.7%)	
Unemployment	271 (94.8%)	120 (89.6%, 44.3%)	151 (99.3%, 55.7%)	
**Residence**				0.0298
Community	242 (84.6%)	120 (89.6%, 49.6%)	122 (80.3%, 50.4%)	
Institution	44 (15.4%)	14 (10.5%, 31.8%)	30 (19.7%, 68.2%)	
**Urbanization level**				0.4294
Rural	30 (10.5%)	16 (11.9%, 53.3%)	14 (9.2%, 46.7%)	
Suburban	91 (31.8%)	46 (34.3%, 50.6%)	45 (29.6%, 49.5%)	
Urban	165 (57.7%)	72 (53.7%, 43.6%)	93 (61.2%, 56.4%)	
**ICF category of Disability Types** ^**2**^				0.034
Chapter 1	5 (1.8%)	2 (1.5%, 40.0%)	3 (2.0%, 60.0%)	
Chapter 2	6 (2.1%)	2 (1.5%, 33.3%)	4 (2.6%, 66.7%)	
Chapter 3	6 (2.1%)	4 (3.0%, 66.7%)	2 (1.3%, 33.3%)	
Chapter 4	30 (10.5%)	19 (14.2%, 63.3%)	11 (7.2%, 36.7%)	
Chapter 5	6 (2.1%)	3 (2.2%, 50.0%)	3 (2.0%, 50.0%)	
Chapter 6				
Chapter 7	189 (66.1%)	93 (69.4%, 49.2%)	96 (63.2%, 50.8%)	
Chapter 8				
Multiple Disabilities	44 (15.4%)	11 (8.2%, 25.0%)	33 (21.7%, 75.0%)	
**Disability level**				0.0003
Mild	91 (31.8%)	58 (43.3%, 63.7%)	33 (21.7%, 36.3%)	
Moderate	99 (34.6%)	40 (29.9%, 40.4%)	59 (38.8%, 59.6%)	
Severe	72 (25.2%)	31 (23.1%, 43.1%)	41 (27.0%, 56.9%)	
Extreme	24 (8.4%)	5 (3.7%, 20.8%)	19 (12.5%, 79.2%)	
**Ambulatory Status**				< 0.0001
Ambulatory	162 (56.6%)	97 (72.4%, 59.9%)	65 (42.8%, 40.1%)	
Not ambulatory	122 (42.7%)	36 (26.9%, 29.5%)	86 (56.6%, 70.5%)	
missing	2 (0.7%)	1 (0.8%, 50.0%)	1 (0.7%, 50.0%)	
**Follow-up(years)**	3 (± 1.6)	4 (± 1.6)	3 (± 1.5)	0.0126
1	26 (9.1%)	14 (10.4%)	12 (7.9%)	
2	18 (6.3%)	10 (7.5%)	8 (5.3%)	
3	9 (3.1%)	6 (4.5%)	3 (2.0%)	
4	21 (7.3%)	18 (13.4%)	3 (2.0%)	
5	15 (5.2%)	12 (9.0%)	3 (2.0%)	
missing	197 (68.9%)	74 (55.2%)	123 (80.9%)	

The data are presented as numbers/means (col%, row%/±sd)

Note. 1. Chi-square test

2. Chapter 1 Mental Functions & Structures of the Nervous System,

Chapter 2 Sensory Functions and Pain: The Eye, Ear and Related Structures

Chapter 3 Functions & Structures of/involved in Voice and Speech,

Chapter 4 Functions & Structures of/Related to the Cardiovascular Hematological, Immunological and Respiratory Systems,

Chapter 5 Functions and Structures of/Related to Digestive, Metabolic and Endocrine Systems,

Chapter 6 Functions & Structures of/related to the Genitourinary and Reproductive Systems,

Chapter 7 Neuromusculoskeletal and Movement-related Functions and Structures,

Chapter 8 Functions and Related Structures of Skin

The WHODAS 2.0 domain profiles at the initial evaluation are presented in Table 2. The ≥ 65 years age group scored significantly higher (more severe functional impairments) than did the 18–64 years age group across all domains, with the exception of social participation (*p* = 0.2549), which suggested that the young group may encounter the same level of difficulties in social participation as the elderly individuals. People with MG who indwelled at the institution had significantly greater WHODAS 2.0 scores (more severe functional impairment) than those who lived in the community across all domains regardless of age group. Furthermore, people with multiple MG-associated disabilities (≥ 2 systems involved) had significantly higher WHODAS 2.0 scores across all domains except for social participation (*p* = 0.3082). This may imply that social participation was a more vulnerable functional domain than the other domains. The result also revealed people with MG who preserved ambulatory function had significantly better functional status.

**Table 2:** Initial WHODAS 2.0 scores by group

	Cognition	Mobility	Self-care	Getting along	Life activities	Participation	Summary
	Mean (± SD)	Mean (± SD)	Mean (± SD)	Mean (± SD)	Mean (± SD)	Mean (± SD)	Mean (± SD)
**Age (years)**							
18–64	26.8 (± 26.1)	60.0 (± 32.4)	48.4 (± 35.5)	43.1 (± 33.2)	69.6 (± 33.4)	53.7 (± 27.4)	48.7 (± 25.2)
≥ 65	52.3 (± 32.2)	84.5 (± 22.7)	74.9 (± 28.5)	61.6 (± 33.1)	90.3 (± 18.8)	56.6 (± 23.9)	56.6 (± 23.9)
*p* value^1^	< 0.0001	< 0.0001	< 0.0001	< 0.0001	< 0.0001	0.2549	< 0.0001
**Residence**							
Community	36.8 (± 29.9)	69.9 (± 31.0)	58.1 (± 34.8)	49.5 (± 33.7)	78.0 (± 29.8)	52.3 (± 25.0)	55.0 (± 24.3)
Institution	60.2 (± 36.4)	90.1 (± 17.2)	86.4 (± 20.9	71.6 (± 32.1)	94.7 (± 13.9)	71.9 (± 22.6)	76.8 (± 19.6)
*p* value^1^	< 0.0001	< 0.0001	< 0.0001	< 0.0001	0.0005	< 0.0001	< 0.0001
**Multiple disabilities**							
No	36.7 (± 31.0)	70.4 (± 30.9)	58.7 (± 35.2)	50.3 (± 34.5)	78.4 (± 29.5)	54.6 (± 26.1)	55.9 (± 25.1)
Yes	60.6 (± 30.8)	87.6 (± 20.2)	83.4 (± 21.0)	67.0 (± 30.1)	92.0 (± 19.4)	59.0 (± 22.8)	71.6 (± 18.2)
*p* value^1^	< 0.0001	0.0003	< 0.0001	0.0023	0.0067	0.3082	0.0001
**Ambulatory Status**							
Ambulatory	29.5 (± 27.0)	54.5 (± 28.0)	44.4 (± 32.3)	38.7 (± 31.1)	67.5 (± 31.6)	46.7 (± 24.6)	45.3 (± 22.4)
Not ambulatory	55.2 (± 32.6)	97.1 (± 6.5)	86.5 (± 19.6)	72.0 (± 29.2)	97.7 (± 7.5)	66.6 (± 22.1)	75.8 (± 15.7)
*p* value^1^	< 0.0001	< 0.0001	< 0.0001	< 0.0001	< 0.0001	< 0.0001	< 0.0001

Note: 1. There was a significant difference (*p* < 0.05) between the two age groups

The RD between the WHODAS 2.0 capability and performance scores at the initial evaluation was significantly different across all domains in both groups (Table 3), which suggests that the use of orthosis or environment modification could significantly improve patient function. The WHODAS 2.0 scores did not showed significantly different between the 1st and 2nd assessments (Table 4).

**Table 3:** Comparison of the performance and capabilities of the initial WHODAS 2.0

Domain	18–64 years		65 & 65 years above	
	Differences^1^	*p* value^2^	Differences^1^	*p* value^2^
**Cognition (Domain 1)**	4.1 (± 12.2)	< 0.0001	5.4 (± 9.7)	< 0.0001
**Mobility (Domain 2)**	13.2 (± 17.3)	< 0.0001	20.8 (± 18.9)	< 0.0001
**Self-care (Domain 3)**	18.1 (± 26.5)	< 0.0001	35.1 (± 31.7)	< 0.0001
**Getting along (Domain 4)**	1.8 (± 7.3)	0.0039	4.3 (± 11.5)	< 0.0001
**Life activities (Domain 5 − 1)**	13.7 (± 28.6)	< 0.0001	16.3 (± 31.3)	< 0.0001
**Participation (Domain 6)**	5.1 (± 12.3)	< 0.0001	7.1 (± 12.0)	< 0.0001
**Summary**	8.2 (± 9.5)	< 0.0001	12.7 (± 10.2)	< 0.0001

Note: 1. Differences = Capability - Performance

2. A significant difference (p < 0.05) was determined using the Wilcoxon signed rank test

**Table 4:** Differences of WHODAS 2.0 score of capability between two evaluations

Domain	Differences of WHODAS 2.0 Score			
	18–64 years	*p* value	65 & 65 years above	*p* value
**Cognition (Domain 1)**	1.3 (± 31.4)	0.8366	2.8 (± 33.1)	0.488
**Mobility (Domain 2)**	1.5 (± 27.7)	0.488	1.0 (± 17.5)	0.819
**Self-care (Domain 3)**	0.4 (± 39.9)	0.9256	-4.9 (± 26.2)	0.5175
**Getting along (Domain 4)**	3.7 (± 35.2)	0.819	-0.5 (± 39.6)	0.7878
**Life activities (Domain 5 − 1)**	2.8 (± 29.8)	0.5804	1.1 (± 17.4)	0.8672
**Participation (Domain 6)**	-9.6 (± 27.0)	0.0018	-1.1 (± 31.0)	0.8883
**Summary**	-1.3 (± 23.8)	0.4422	0.0 (± 20.6)	0.9326

Note: 1. Differences = Follow-up score– initial score

2. Significant difference (*p* < 0.05), using Wilcoxon signed rank test

Table 5 showed the results from the crude analysis of GEE to examine associations between functional changes in each WHODAS 2.0 domain and demographic characteristics across the follow-up time. We observed significantly greater functional improvements (decreased WHODAS 2.0 scores) in people with MG who were unable to ambulate at the initial evaluation (decreased 12.55 in the summary scores) and in those who indwelled at the institution (decreased 12.57 in the summary scores). Among included variables, having Chap. 4 disability (respiratory system involvement) was a significant factor associated with cognitive decline (increased 18.03 in cognition domain). The functional changes between sex, age and disability groups (with one vs. with multiple disability) did not significantly differ across all domains. After adjusting for confounders, the results showed that there were no significant differences in functional changes between age groups (< 65 years vs. ≥ 65 years) across all domains [*p* = 0.7854, 95% CI (-8.43-11.16)]. These findings suggest that young people may encounter the same level of change in functioning as elderly people. Furthermore, having Chap. 4 disability was significantly associated with cognitive decline. [*p* = 0.0435, 95% CI (0.44–29.28)] (Table 6).

**Table 5:** GEE crude analysis for predicting the changes of the WHODAS 2.0 scores across the Follow-up time between variables

Variables	Capability						
	Cognition	Mobility	Self-care	Getting along	Life activities	Participation	Summary
Sex (Ref = Male)	-5.39	-4.30	-10.13	-8.74	0.94	1.91	-2.99
Age (Ref = < 65 years)	4.14	-1.81	-10.12	1.13	0.04	6.32	1.04
Residence (Ref = Community)	-6.15	-10.28*	-19.34	-9.31	-9.00	-19.36*	-12.57*
Urban level (Ref = Urban)	10.68	4.07	7.36	14.55*	-0.89	2.39	5.09
Chapter 4 Disability (Ref = No)	18.03*	15.35*	24.41*	17.99*	20.79*	8.22	16.77*
Multiple Disabilities (Ref = No)	2.44	-2.55	-13.34	-2.53	-10.60	-3.03	-3.71
Ambulatory Status (Ref = Ambulatory)	-5.01	-15.41*	-17.28*	-21.74*	-12.14*	-10.85	-12.55*

Note: * *p* < 0.05, ** *p* < 0.0001; GEE = generalized estimating equations; Ref = reference

**Table 6:** GEE multivariate linear regression model for predicting the changes of the WHODAS 2.0 scores across the Follow-up time between variables

Main effects	Cognition	Mobility	Self-care	Getting along	Life activities	Participation	Summary
	β (P-value)	β (P-value)	β (P-value)	β (P-value)	β (P-value)	β (P-value)	β (P-value)
**Time**							
T0	Ref	Ref	Ref	Ref	Ref	Ref	Ref
T1	-1.14 (0.8091)	66.09 (< 0.0001)	53.08 (< 0.0001)	45.76 (< 0.0001)	71.13 (< 0.0001)	55.79 (< 0.0001)	51.91 (< 0.0001)
**Sex**							
Female	Ref	Ref	Ref	Ref	Ref	Ref	Ref
Male	-2.45 (0.5041)	-0.24 (0.9538)	0.2 (0.9715)	2.77 (0.585)	-1.13 (0.7701)	-8.71 (0.0053)	-2.29 (0.4832)
**Age**,** years**							
18–64	Ref	Ref	Ref	Ref	Ref	Ref	Ref
≥65	21.02 (0.0002)	9.08 (0.0089)	-6.56 (0.0946)	-2.97 (0.4702)	-2.72 (0.4344)	-3.07 (0.3353)	-4.34 (0.1397)
**RD**							
No	Ref	Ref	Ref	Ref	Ref	Ref	Ref
Yes	-11.65 (0.0164)	23.95 (< 0.0001)	22.22 (0.0003)	15.29 (0.0127)	15.92 (0.0005)	-0.42 (0.9335)	14.76 (0.0009)
**Education**,** years**							
>6	Ref	Ref	Ref	Ref	Ref	Ref	Ref
≤6	3.37 (0.5488)	-18.39 (0.0019)	-15.11 (0.0172)	-11.53 (0.0652)	-8.98 (0.0926)	-6.99 (0.1701)	-11.69 (0.01)
**Interaction effects**							
Time ⊆ Sex	-5.89 (0.3593)	0.23 (0.9559)	3.44 (0.5743)	2.72 (0.6490)	5.8 (0.1738)	3.62 (0.4706)	3.14 (0.466)
Time ⊆ Age	4.86 (0.4849)	-3.86 (0.4585)	-8.96 (0.1914)	-8.94 (0.2034)	1.1 (0.8347)	1.76 (0.7568)	-3.09 (0.5222)
Time ⊆ RD	14.5 (0.0408)	-1.66 (0.7227)	-9.26 (0.1821)	2.22 (0.7724)	-0.39 (0.9358)	6.28 (0.3207)	1.27 (0.7916)

Abbreviations: T0: initial; T1: follow-up; GEE = generalized estimating equations. Ref = reference; RD = Respiratory Dysfunction

## Discussion

To our knowledge, this is the first large-scale analysis with a long-term follow-up on functional disabilities in people with MG. Approximately 5,500 individuals were living with MG in Taiwan during the study period [18]. Among them, 286 individuals with MG-associated disabilities were included in our study. This suggests that approximately one in every 20 individuals with MG experiences substantial disability. We found that the dysfunction of social participation in the 18–64 years age group did not significantly differ from that in the ≥ 65 years age group, suggesting that MG could affect patients’ abilities to fulfill family and social roles and act as barriers to obtaining employment as early as working age. Indeed, of our participants, only 10.5% were employed in the group aged 18–64 years. Studies have demonstrated that MG is associated with an increased risk of long periods of unemployment [19, 20]. In addition, this study revealed that people with only one MG-associated disability had better functional performance than patients with multiple disabilities, except in the domain of social participation. This underscores the vulnerability of social participation in MG patients, suggesting the need for a multidisciplinary approach to address this aspect, including supported community engagement and occupational rehabilitation, despite people with milder disabilities.

In our cohort, the provision of technical aids or other environmental interventions resulted in significant functional improvements across all domains. This finding aligns with previous review study indicating that rehabilitative modalities, including physical training, respiratory training, and balance training, contribute to enhancing the quality of life of MG patients [21]. In our follow-up analyses, no significant differences were identified in the level of functional improvement between the sex, age and disability (with one vs. with multiple disability) groups, emphasizing the importance of adopting environmental support and assistive devices for all MG patients, irrespective of disability severity or age, to mitigate functional impairments.

At follow-up, we observed significantly greater functional improvements in people with MG who were unable to ambulate independently, without respiratory dysfunctions (Chap. 4 disability), and who were indwelled at the institution. This finding is in line with our previous study involving a five-year follow-up of patients with stroke, which demonstrated that those requiring assistance with ambulation experienced significant improvements across most WHODAS 2.0 functional domains [22]. Such improvements may be attributed to greater access to rehabilitation services, likely facilitated by National Health Insurance coverage based on the level of disability.

In the early stage, the MG mostly affects the muscles that control eye movement, facial expression, and swallowing. As the condition progresses, limb muscles may also be affected, causing difficulty walking. Despite improvements in the medical management of MG in recent years, patients continue to report poor well-being outcomes, such as reduced quality of life, walking limitations and decreased balance. In our study, patients with myasthenia gravis (MG) who were unable to walk independently at the initial evaluation demonstrated greater functional improvement at follow-up compared to those in the ambulatory group. This finding is consistent with our previous study using the same database, in which ambulatory status was identified as an independent determinant of functional outcomes in patients with Parkinson’s disease [23]. MG patients can be classified into subgroups based on the localization of muscle weakness, thymic pathology, and age at onset. These subgroups likely differ in their response to physical training and exercise, depending on the underlying pathogenesis. Rehabilitation plays a critical role, particularly in MG patients with gait disturbances, by improving energy efficiency and enhancing compatibility with orthotic devices [24]. Evidence has shown that increased physical activity is associated with favorable outcomes [25]. Therefore, patients with difficulty walking may be encouraged to engage in rehabilitation to restore functioning. Specialized exercise training, including body weight-supported treadmill ambulation, robotic-assisted gait training, and neuromuscular electrical stimulation, is beneficial for improving walking speed in patients with MG [26]. On the other hand, patients with MG who still have preserved ambulatory function might overlook the importance of exercise intervention. A previous study showed that more functional individuals with MG were more inactive and performed less physical activity [27]. A review article including ten intervention studies revealed that individualized physical training and exercise are safe for individuals with MG [24]. Tailored exercise programs and lifestyle modifications, such as reducing sedentary time, are important for people with MG.

The course of myasthenia gravis (MG) is complicated by increased reports of cognitive defects such as lower memory performance in both immediate and delayed recall. This trend implies potential damage to the central nervous system (CNS) [28]. Cognitive impairment might not be directly correlated with the length or severity of MG [29]. A previous study revealed several independent factors associated with cognitive impairment in patients with MG, including advanced age, diabetes, and thyroid dysfunction [30]. In our study, patients with Chap. 4 disabilities (disabilities involving respiratory systems) showed greater cognitive decline. This finding may suggest that respiratory dysfunction is a significant determinant of cognitive decline in people with MG. Weakness in the respiratory muscles represents the most severe manifestation of MG. In addition. a potential mechanistic link has been proposed, suggesting that respiratory issues could be intertwined with the detection of centrally acting acetylcholine receptor (AchR)-antibodies, cytokines, and hypoxia, ultimately culminating in impaired cognitive function [31]. Several studies have shown that muscle endurance training combined with breathing retraining is beneficial for improving aerobic capacity, increasing forced vital capacity and reducing respiratory complications in people with MG [32, 33]. 

Studies have suggested that MG was negatively correlated with labor market participation, with 20% of patients with MG being reported to have stopped working prior to retirement age [34]. An Australian study demonstrated that more than 20% patients with MG stopped working or reduced their working hours due to the disease [35]. A German study of 1518 patients with MG revealed that nearly 70% had no labor market participation [36]. Previous meta-analysis study also suggested the proportion of employed MG patients varied from 28 to 82%, with an extreme heterogeneity between studies [37]. The reduced employment rate in our study may be attributable to the inclusion of individuals with MG who also had substantial disabilities, as well as to differences in labor market policies across countries. Furthermore, homemakers, students, and volunteers were not classified as employed, which may have led to an underestimation of the true employment rate. Beyond physical limitations, generalized fatigue, concomitant autoimmune conditions, and treatment side effects may further impair the ability to maintain employment, generalized fatigue, concomitant autoimmune diseases, and side-effects from treatment may negatively influence employment [38, 39]. In some patients with MG, impaired facial expressions and communication difficulties may also hinder work participation [40]. Future research may focus on return-to-work interventions among patients with MG to optimize their work participation since participating in the labor force is a key facilitator of social participation among those with chronic health conditions.

## Strengths and limitations

This study has the following strengths. First, we used the WHODAS 2.0, a well-validated, objective measure of functional impairment, which enabled us to comprehensively analyze the functional limitations of MG. The WHODAS 2.0 has also been used to assess disability progression in other rare neurological diseases, such as amyotrophic lateral sclerosis and multiple sclerosis [41, 42]. Accordingly, our use of internationally recognized scales may increase the generalizability of our findings. Moreover, demographic data were collected from a large nationally representative database covering the entire spectrum of basic and disability characteristics. The limitation of study are the inclusion criteria focused solely on patients with MG who had registered disabilities. Consequently, our findings may overrepresent individuals with more severe forms of the disease. In addition, we could not obtain information on the subtypes of MG for all participants. However, the sex ratio and the average age of our participants were comparable to those in other population studies of MG [43, 44]. The variable follow-up period, ranging from 1 to 5 years, with 68.9% of data lost, which may also significantly impact the conclusions drawn. Finally, the database used in this study did not include information on disease duration, treatment status, or comorbidities, which may have acted as confounding factors in our analysis. Overall, our results revealed that although a decrease in disability level occurred after environmental intervention, significant functional impairment was still noted in multiple dimensions in people with MG. Moreover, even patients who were younger and had milder disabilities exhibited vulnerability to social participation dysfunction. Clinical practitioners are encouraged to identify risk factors contributing to social challenges in these patients, facilitating timely management.

## Conclusions

Our nationwide study revealed a broad spectrum of functional disabilities among individuals with MG, highlighting social participation as a particularly vulnerable domain. Respiratory disability, in particular, was found to be a significant contributor to cognitive decline. Environmental modifications and assistive devices may benefit all patients, regardless of age. Although collecting specific data on interventions and disease subtypes can be challenging—particularly in retrospective or national database studies—this study provides valuable insights into the relationships between functional changes and other outcomes over time. These findings enhance our understanding of potential risk factors for disability in MG and highlight the need for prospective studies to determine whether targeted rehabilitation could help prevent disability progression.

## Data Availability

The data associated with the paper are not publicly available due to the need to protect patient privacy but are available from the corresponding author on reasonable request.

## Abbreviations

AchR: Acetylcholine receptor
CNS: Central nervous system
GEE: Generalized estimating equation
ICF: International Classification of Functioning, Disability, and Health
MG: Myasthenia gravis
NMJ: Neuromuscular junction
RDs: Relative differences
TDPD: Taiwan Data Bank of Persons with Disability
WHODAS 2.0: WHO Disability Assessment Schedule 2.0
